# Comparison between peak systolic velocity of the inferior thyroid artery and technetium-99m pertechnetate thyroid uptake in differentiating Graves’ disease from thyroiditis

**DOI:** 10.20945/2359-3997000000165

**Published:** 2019-08-28

**Authors:** Sajad Ahmad Malik, Naseer Ahmad Choh, Raiz Ahmad Misgar, Shoukat H. Khan, Zaffar A. Shah, Tanveer Ahmad Rather, Faheem Shehjar, Bashir Ahmad Laway

**Affiliations:** 1 Department of Endocrinology SKIMS Srinagar India Department of Endocrinology, SKIMS, Srinagar, India; 2 Department of Radiology SKIMS Srinagar India Department of Radiology, SKIMS, Srinagar, India; 3 Department of Nuclear Medicine SKIMS Srinagar India Department of Nuclear Medicine, SKIMS, Srinagar, India; 4 Department of Immunology SKIMS Srinagar India Department of Immunology, SKIMS, Srinagar, India

**Keywords:** Color-flow Doppler, Graves’ disease, thyroiditis, technetium thyroid uptake, TSH-receptor antibody

## Abstract

**Objectives:**

The differentiation between the various etiologies of thyrotoxicosis, including those with hyperthyroidism (especially Graves’ disease [GD], the most common cause of hyperthyroidism) and without hyperthyroidism (like thyroiditis), is an important step in planning specific therapy. Technetium-99m (^99m^Tc) pertechnetate thyroid scanning is the gold standard in differentiating GD from thyroiditis. However, this technique has limited availability, is contraindicated in pregnancy and lactation, and is not helpful in cases with history of recent exposure to excess iodine. The aim of this study was to identify the diagnostic value of the peak systolic velocity of the inferior thyroid artery (PSV-ITA) assessed by color-flow Doppler ultrasound (CFDU) and compare the sensitivity and specificity of this method versus ^99m^Tc pertechnetate thyroid uptake.

**Subjects and methods:**

We prospectively analyzed 65 patients (46 with GD and 19 with thyroiditis). All patients were evaluated with clinical history and physical examination and underwent ^99m^Tc pertechnetate scanning and measurement of TRAb levels and PSV-ITA values by CFDU. The diagnosis was based on findings from signs and symptoms, physical examination, and ^99m^Tc pertechnetate uptake.

**Results:**

Patients with GD had significantly higher mean PSV-ITA values than those with thyroiditis. At a mean PSV-ITA cutoff value of 30 cm/sec, PSV-ITA discriminated GD from thyroiditis with a sensitivity of 91% and specificity of 89%.

**Conclusion:**

Measurement of PSV-ITA by CFDU is a good diagnostic approach to discriminate between GD and thyroiditis, with sensitivity and specificity values comparable to those of ^99m^Tc pertechnetate thyroid uptake.

## INTRODUCTION

Thyrotoxicosis is characterized by exposure of the body to excessive thyroid hormone levels, commonly due to a release of high levels of thyroid hormones from the thyroid gland. This may occur from increased thyroid functioning (hyperthyroidism) or inflammatory conditions such as subacute or Hashimoto’s thyroiditis. Hyperthyroidism can occur due to Graves’ disease (GD), toxic multinodular goiters, or solitary toxic nodules (1). The prevalence of thyrotoxicosis in India is currently unclear.

The differentiation between the various etiologies of thyrotoxicosis, including those with hyperthyroidism (especially GD, the most common cause of hyperthyroidism) and without hyperthyroidism (like thyroiditis), is an important step in planning specific therapy. In the absence of specific signs of GD, such as eye, skin, and nail changes, the diagnosis of this condition may be difficult, and evaluation with complementary tests becomes necessary (2). Although technetium 99m (^99m^Tc) pertechnetate scanning is frequently used to differentiate GD from thyroiditis, its limited availability and some contraindications (such as pregnancy and lactation) have prompted us to use other diagnostic modalities like color-flow Doppler ultrasound (CFDU). Additionally,^99m^Tc pertechnetate scanning is not helpful in patients with recent exposure to excessive iodine in the form of iodine-containing drugs, food, and contrast (3).

Interest in CFDU as an important tool in the differential diagnosis of thyrotoxicosis has grown in recent times. Echogenic features and vascular pattern can both differentiate hyperthyroidism from thyroiditis (4-6). However, the echogenicity of the thyroid is decreased in most etiologies of thyrotoxicosis (including GD, subacute thyroiditis, and Hashimoto’s thyroiditis) and there is considerable overlap in terms of echogenicity between GD and thyroiditis (7-9). Increased vascular pattern on CFDU is an important feature of GD and helps differentiate this condition from thyroiditis. However, assessment of the vascular pattern is a subjective finding and is difficult to quantify (2,4,9-12). Detection of peak systolic velocity (PSV) of either the superior or inferior thyroid artery (ITA) is a quantitative measurement. Increased PSV of the superior or inferior thyroid arteries is a more specific and sensitive finding in GD and is absent in thyroiditis (3,10-12).

The comparison between measurement of PSV of the ITA (PSV-ITA) by CFDU versus ^99m^Tc pertechnetate thyroid scanning and TRAb levels in the Asian population still lacks data. Based on these considerations, the aim of this study was to assess the role of PSV-ITA measurement by CFDU in the differential diagnosis of GD and subacute thyroiditis and to compare the sensitivity and specificity values of PSV-ITA with those of ^99m^Tc pertechnetate thyroid scan uptake in subjects with GD and thyroiditis.

## SUBJECTS AND METHODS

The study was approved by the hospital’s Ethics Committee and included 65 treatment-naïve patients with thyrotoxicosis attending an Endocrinology outpatient facility at a tertiary care center in North India between February 2015 and July 2016. Each patient was evaluated according to a predefined protocol. We collected information regarding the patients’ age, gender, and signs and symptoms of thyrotoxicosis. The thyroid was examined in terms of size, consistency, tenderness, and presence of nodules and bruit. The occurrence of ophthalmopathy and dermopathy was investigated. The diagnosis of GD was established based on clinical features of thyrotoxicosis, presence of diffuse goiter with or without eye, skin or nail changes, and diffusely increased ^99m^Tc pertechnetate uptake on thyroid scintigraphy. In a few patients, the diagnosis of GD was confirmed by the presence of positive TRAb levels and follow-up findings. The reference values of ^99m^Tc thyroid uptake at 15 minutes (0.4 to 7%) were retrieved from ethnic-matched, normal controls participating in a previous study (13). The diagnosis of thyroiditis was based on the presence of thyroid tenderness, absence of eye, skin or nail changes, absence of positive TRAb levels, decreased ^99m^Tc uptake, and development of spontaneous euthyroidism or hypothyroidism during follow-up. We excluded from the study those patients with a previous history of thyroid surgery, exposure to radioiodine therapy, pregnancy, and lactation, as well as individuals taking medications containing iodine (including amiodarone) and recent exposure to iodine-containing contrast agents.

### Laboratory tests

Blood samples were collected from each patient during fasting. Serum concentrations of TSH, total T3, and total T4 were measured by commercial chemiluminescent immunoassays (Beckman Coulter Unicel DXI 800 Access Immunoassay System, Brea, California, USA). TRAb levels were measured by enzyme-linked immunosorbent assay (ELISA; Elisa RSR TRAb 2nd generation kit, RSR Ltd, Cardiff, UK). The reference values are 0.5–6.5 mIU/mL for TSH, 0.70–2.50 ng/mL for total T3, 4.0–13.0 μg/dL for total T4, and ≤ 1 U/L for TRAb.

### TRAb measurement

For measurement of TRAb levels, we added 75 µL of buffer solution to each TSHR-coated well, followed by 75 µL of test sera, calibrators, and controls. The wells were then covered with a lid and shaken for 2 hours at room temperature on an ELISA plate shaker (500 shakes/min). After washing with wash solution, 100 µL of reconstituted TSH-biotin was added to each well and then incubated at room temperature for 25 minutes. After another wash, 100 µL of diluted streptavidin-peroxidase (SA-POD) was added and again incubated at room temperature for 20 minutes without shaking. This was followed by another washing with wash solution and then pure water. After that, 100 µL of 3, 3’, 5, 5’-tetramethylbenzidine (TMB) was added into each well and incubated in the dark at room temperature for 30 minutes. After this step, additional reaction was stopped by adding 50 µL of stop solution to each well and, after covering with a lid, the wells were shaken for approximately 5 seconds on an ELISA plate shaker. The absorbance of each well was then read at 450 nm using an ELISA plate reader. A calibration curve was established by plotting the calibrator concentration on the x-axis (log scale) against the absorbance of the calibrators on the y-axis (linear scale). Serum TRAb concentrations were then read from the calibration curve. The cutoff level considered for the diagnosis of GD was > 1 IU/L, while values ≤ 1 IU/L were diagnostic of thyroiditis, as indicated by the manufacturer of the TRAb kit.

### Evaluation with color-flow Doppler ultrasound

The study participants underwent conventional ultrasonography and CFDU using a 7.5 MHz broadband linear transducer (Aloka ProSound 3500 SX, Hitachi Aloka Medical, Ltd., Tokyo, Japan) by an experienced radiologist who was blinded to the patients’ clinical and biochemical parameters. The observed parameters included the size, shape, echo pattern, presence or absence of nodules, vascular pattern (“thyroid inferno”), and PSV of both the right and left ITAs. The patients were examined in dorsal decubitus with a cushion under their shoulders and neck extended. The probe was positioned softly without compressing the skin to prevent underestimation of the vascular intensity. The ITA was examined in the transverse plane at the junction of the middle and inferior third of the thyroid. The cursor was maintained close to the trachea to avoid artifacts from the common carotid artery and internal jugular vein. The PSV-ITA was obtained from both the right and left sides. The Doppler angle was corrected to values under or equal to 60°. The mean PSV-ITA value found in the right and left lobes was used as a representative parameter.

### Technetium (^99m^Tc) pertechnetate thyroid scintigraphy

^99m^Tc pertechnetate thyroid scans were evaluated by two nuclear medicine physicians who were blinded to the CFDU findings. The parameters observed included the uptake pattern and percentage, and the uptake by the salivary glands. For the ^99m^Tc pertechnetate thyroid scans, 15 minutes after intravenous injection of ^99m^Tc pertechnetate 185 MBq, static images were obtained using a large field-of-view dual-head gamma camera fitted with a low-energy all-purpose parallel-hole collimator (dual-head, E.cam, Siemens Medical Solutions, Hoffman Estates, IL, USA) with the patient in a supine position with extended neck. The anterior, left anterior oblique, and right anterior oblique view images were recorded with a 128 x 128 matrix size and zoom set to 1.0. The energy level of the gamma camera was set at 140 keV, with a 20% symmetrical window. Background- and decay-corrected thyroid uptakes were calculated using a predefined software provided with the system.

### Statistical analysis

The statistical analysis was performed using the SPSS Statistical Package, version 16.0 (SPSS Inc., Chicago, IL, USA). The data are expressed as mean ± standard deviation. We used the chi-square test for categorical data and the independent samples Student’s *t* test for parametric quantitative data. Pearson’s correlation test was used to compare parametric quantitative data. We used receiver operating characteristic (ROC) curve analysis to obtain optimum cutoff values of PSV-ITA and TRAb.

## RESULTS

All 65 patients had suppressed TSH levels and increased total T3 and T4 levels. Overall, 46 patients had GD and 19 patients had thyroiditis. A T3/T4 ratio below 20 was present in 18 (95%) patients with thyroiditis and 31 patients (67%) with GD. This difference in T3/T4 ratio between both groups was statistically significant (*p* = 0.02). Patients with GD had larger thyroid volumes than those with thyroiditis (17.86 ± 8.12 cm^3^ versus 7.36 ± 2.6 cm^3^, respectively, *p* < 0.001) (Table 1).

**Table 1:** Demographic and baseline characteristics of the study participants

Parameter	Graves’ disease (n = 46)	Thyroiditis (n = 19)	*P* value
Sex, number (%)			0.256
Male	17 (37)	4 (21)	
Female	29 (63)	15 (79)	
Age in years, mean (SD)	35.26 (13.06)	31.21 (8.87)	0.221
Total T4, μg/dL	20.42 (4.89)	22.25 (5.04)	0.181
Total T3, ng/mL	4.07 (1.49)	2.55 (0.62)	< 0.001
TSH, mIU/L	0.03 (0.05)	0.02 (0.02)	0.58
T3 to T4 ratio < 20, value (%)	31 (67)	18 (95)	0.02
T3 to T4 ratio ≥ 20, value (%)	15 (33)	1 (5)	0.02

SD: standard deviation.

Patients with GD had higher mean PSV-ITA values than those with thyroiditis (45.85 ± 14.49 versus 15.83 ± 8.15, respectively, *p* < 0.001). A ROC curve analysis of the mean PSV-ITA values yielded an area under the curve (AUC) of 0.97, with a cutoff value of 30 cm/sec emerging as an appropriate value to differentiate between GD and thyroiditis (sensitivity 91%, specificity 89%). The percentage ^99m^Tc pertechnetate uptake was 43.05 ± 0.77 in patients with GD and 2.57 ± 6.33 in those with thyroiditis (*p* < 0.001). TRAb levels were more markedly elevated in patients with GD compared with those with thyroiditis (*p* < 0.001) (Table 2).

**Table 2:** Diagnostic ability of TRAb, mean PSV-ITA, and 99mTc pertechnetate thyroid scanning in the differential diagnosis of thyrotoxicosis

TRAb levels	mPSV-ITA	^99m^Tc pertechnetate uptake
AUC 0.968	0.976	0.985
95% CI 0.906-1.03	0.940-1.01	0.957-1.01
Cutoff value1.5 U/L	30 cm/sec	7%
Sensitivity (%) 100	91	100
Specificity (%) 89	89	84
PPV (%) 96	95	94
NPV (%) 100	80	100
Diagnostic 97 accuracy (%)	91	95

AUC: area under the curve; CI: confidence interval; NPV: negative predictive value; PPV: positive predictive value; mPSV-ITA: mean peak systolic velocity of the inferior thyroid artery; ^99m^Tc = technetium-99m.

We found a significant correlation between PSV-ITA values and serum total T3 levels (r = 0.52, *p* = 0.001. We also found a significant positive correlation between ^99m^Tc pertechnetate uptake and PSV-ITA values (r = 0.72, *p* = 0.001, and a strong correlation between TRAb levels and PSV-ITA values (r = 0.40, *p* < 0.001).

## DISCUSSION

The differentiation between GD and thyroiditis can be challenging, especially in mild GD cases that are not associated with typical features of the disease, like ophthalmopathy or dermopathy, although the persistence of signs and symptoms is helpful in differentiating GD from thyroiditis. For proper management, the disease must be diagnosed early. Even though radioactive iodine or ^99m^Tc pertechnetate uptake is the gold standard in the diagnosis of hyperthyroidism, the widespread use of this technique is limited. Also, nuclear scanning is not available in many facilities, is contraindicated in pregnancy and lactation, and is affected by recent iodine exposure. The strength of the present study is the use of multiple modalities for the diagnosis of GD, which in addition to nuclear scanning included measurement of TRAb levels. In contrast, the ultrasonographic evaluations were carried out by a single ultrasonographer, which is a limitation of the study.

Measurement of biochemical parameters like thyroid hormone levels have been suggested to help identify the etiology of the thyrotoxicosis (14), and a T3/T4 ratio below 20 has been proposed to indicate thyroiditis (15,16). In the present study, a T3/T4 ratio below 20 was present in 94% of the patients with thyroiditis but was also seen in 69% of those with GD. This indicates a marked overlap between the two conditions when the T3/T4 ratio is used as a diagnostic criterion, which is in agreement with some studies (17).

In the present study, we evaluated the thyroid blood flow as a marker to identify different types of thyrotoxicosis. The mean PSV-ITA value was significantly higher in patients with GD compared with those with thyroiditis. In the present study, the PSV-ITA cutoff value of 30 cm/sec, measured by CFDU, revealed a sensitivity of 91% and a specificity of 89% to diagnose GD. This parameter could help differentiate cases of GD and thyroiditis with equivocal Doppler patterns (9,18). Measurement of PSV appears to be more useful than the evaluation of CFDU patterns in the differential diagnosis of thyrotoxicosis. Our results are in agreement with those of Kurita and cols. (19), who demonstrated a sensitivity of 84% and specificity of 90% for CFDU in the differential diagnosis of thyrotoxicosis. Similarly, Donkol and cols. (2), in a study with 26 patients with thyrotoxicosis, demonstrated a sensitivity of 88.9% and specificity of 87.5% with CFDU, using^99m^Tc pertechnetate as the gold standard. On the other hand, Hari Kumar and cols. (11), in a study of 65 patients with thyrotoxicosis, found a significantly higher ITA blood flow in patients with GD than in those with thyroiditis. The authors demonstrated that CFDU had a sensitivity of 96% and specificity of 95% in establishing a differential diagnosis between GD and thyroiditis in patients with thyrotoxicosis, although their sensitivity and specificity values were higher than those found in our study. Their study was also limited by the facts that the diagnosis was mainly based on clinical signs and symptoms of thyrotoxicosis and that ^99m^Tc pertechnetate scans were not available in most patients.

In our study, ^99m^Tc pertechnetate scanning had a better performance than measurement of PSV-ITA values in terms of sensitivity (100% versus 91%, respectively) and negative predictive value (100% versus 80%, respectively) but both diagnostic modalities had similar specificity and positive predictive values. PSV-ITA may be recommended during pregnancy or lactation, in patients with recent iodine exposure, and in the absence of a nuclear medicine facility. This method is also useful in cases requiring an immediate therapeutic decision, as in cases of severe thyrotoxicosis and patients residing far away.

In our study, three patients with increased ^99m^Tc pertechnetate uptake (*i.e.,* suggestive of GD) were shown to have thyroiditis during follow-up (because of transient thyrotoxicosis). These patients were correctly diagnosed by CFDU as having thyroiditis, due to decreased mean PSV-ITA values. Cases of resolving thyroiditis can occasionally show increased uptake on nuclear imaging, which is similar to the finding in GD, potentially leading to confusion (20).

Other methods of quantitative thyroid blood flow assessment have recently been described by other investigators (21-23). Saleh and cols. (21) performed CFDU on 24 patients with GD and 13 patients with diffuse toxic goiter. In addition to PSV measurement, these authors also measured color pixel density (CPD) and volume flow rate (VFR) and found that PSV had a sensitivity of 87% and specificity of 100% to diagnose GD compared with CPD and VFR measurements, both of which had similar sensitivity and lower specificity (92%) values. Similarly, Kurita and cols. (22) measured the thyroid blood flow area (TBFA) to differentiate between GD and destruction-induced thyrotoxicosis and found a sensitivity of 84% and specificity of 90% for TBFA values between 7.7% and 8%. Ota and cols. (23) examined the role of advanced dynamic flow in differentiating between untreated GD and subacute thyroiditis. The authors concluded that a thyroid blood flow above 4% indicates GD. Despite the use of some new imaging techniques, measurement of PSV with CFDU has sufficient sensitivity and specificity to differentiate GD from thyroiditis.

Using ROC curve, we found that the mean PSV-ITA cutoff value of 30 cm/sec differentiated GD from thyroiditis. Similar cutoff values have been recently published (12). Bogazzi and cols. (18) found that the mean PSV cutoff value of 15 ± 3 cm/sec appropriately diagnosed GD. Many other studies have considered higher cutoff values, probably due to differences in the ethnicity of the study participants and the choice of thyroid artery (superior or inferior) used for the measurement (2,3,24,25).

Thyroid hormone levels have a marked influence on the gland’s vascularity. We found a significant correlation between PSV-ITA values and serum total T3 levels (r = 0.52, 0.001). A similar positive correlation has been observed between free T3 levels and PSV-ITA values by Zuhur and cols. (12). Kumar and cols. also found higher PSV-ITA values in hyperthyroid compared with euthyroid patients with GD (26). Additionally, we found a significant positive correlation between ^99m^Tc pertechnetate uptake and PSV-ITA values (r = 0.72, *p* = 0.001), a finding consistent with that by Zuhur and cols. (12). A study from China has also shown a good correlation between radioactive iodine uptake at 3 and 24 hours and PSV values of the superior thyroid artery (3). We also found a strong correlation between TRAb and PSV-ITA values (r = 0.40, *p* < 0.001). Similarly, Ueda and cols. (27) and Baldini and cols. (28) found a significant correlation between TRAb levels and PSV-ITA values in patients with relapsing GD.

In conclusion, the results of the present study demonstrate that PSV-ITA is as effective as nuclear imaging in the differential diagnosis between GD and thyroiditis. PSV-ITA can be performed on a single day and in patients with contraindications for nuclear scanning (especially during pregnancy and lactation). In contrast to ^99m^Tc pertechnetate thyroid scanning, PSV-ITA is not affected by recent iodine exposure.

## References

[1] Kittisupamongkol W. Hyperthyroidism or thyrotoxicosis? Cleve Clin J Med. 2009;76(3):152. 10.3949/ccjm.76c.03001 19258460

[2] Donkol RH, Nada AM, Boughattas S. Role of color Doppler in differentiation of Graves’ disease and thyroiditis in thyrotoxicosis. World J Radiol. 2013;5(4):178-83. 10.4329/wjr.v5.i4.178 PMC3647210 23671754

[3] Chen L, Zhao X, Liu H, Wang Y, Li L, Lu B, et al. Mean peak systolic velocity of the superior thyroid artery is correlated with radioactive iodine uptake in untreated thyrotoxicosis. J Int Med Res. 2012;40(2):640-64. 10.1177/147323001204000226 22613425

[4] Arslan H, Unal O, Algün E, Harman M, Sakarya ME. Power Doppler sonography in the diagnosis of Graves’ disease. Eur J Ultrasound. 2000;11(2):117-22. 10.1016/s0929-8266(99)00079-8 10781659

[5] Höfling DB, Cerri GG, Juliano AG, Marui S, Chammas MC. Value of Thyroid Echogencity in the Diagnosis of Chronic Autoimmune Thyroiditis. Radiologia Brasileira. 2008;41(6):409-17.

[6] Schiemann U, Gellner R, Riemann B, Schierbaum G, Menzel J, Domschke W, Hengst K. Standardized grey scale ultrasonography in Graves’ disease: correlation to autoimmune activity. Eur J Endocrinol. 1999;141(4):332-6. 10.1530/eje.0.1410332 10526244

[7] Espinasse P. Thyroid echography in chronic autoimmune lymphocytic thyroiditis. J Radiol. 1983;64(10):537-44. 6689341

[8] Gutenkust R, Hafermann W, Mansky T, Scribav PC. Ultrasonography related to clinical and laboratory findings in lymphocytic thyroiditis. Acta Endocrinol. 1989;121(1):129-35. 10.1530/acta.0.1210129 2662693

[9] Vitti P, Rago T, Mazzeo S, Brogioni S, Lampis M, De Liperi A, et al. Thyroid blood flow evaluation by color-flow Doppler sonography distinguishes Graves’ disease from Hashimoto’s thyroiditis. J Endocrinol Invest. 1995;18(11):857-61. 10.1007/BF03349833 8778158

[10] Ralls PW, Mayekawa DS, Lee KP, Colletti PM, Radin DR, Boswell WD, et al. Color-flow Doppler sonography in Graves’ disease: “thyroid inferno”. AJR Am J Roentgenol. 1988;150(4):781-84. 10.2214/ajr.150.4.781 3279732

[11] Hari Kumar KV, Pasupuleti V, Jayaraman M, Abhyuday V, Rayudu BR, Modi KD. Role of thyroid Doppler in differential diagnosis of thyrotoxicosis. Endocr Pract. 2009;15:6-9. 10.4158/EP.15.1.6 19211390

[12] Zuhur SS, Ozel A, Kuzu I, Erol RS, Ozcan ND, Okcan B, et al. The Diagnostic Utility of Color Doppler Ultrasonography, Tc-99m Pertechnetate Uptake and TSH Receptor Antibody for Differential Diagnosis between Graves’ Disease and Silent Thyroiditis: A Comparative Study. Endocr Pract. 2014;20(4):310-9. 10.4158/EP13300.OR 24246346

[13] Khan SH, Mahajan A, Laway BA, Rasool R, Rather TA. Technetium-99m Thyroid Scintigraphy and HLA B-35 in Sub-Acute Thyroiditis-Prospective follow up study from an ethnic Himalayan population of North India- Indian J Nucl Med. 2018;33(4):1-7. 10.4103/ijnm.IJNM_74_18 PMC6194771 30386052

[14] Yanagisava T, Sato K, Kato Y, Shimizu S, Takano K. Rapid differential diagnosis of Graves’ disease and painless thyroiditis using T3/T4 ratio, TSH and total alkaline phosphatase activity. Endocr J. 2005;52(1):29-36. 10.1507/endocrj.52.29 15758555

[15] Amino N, Yabu Y, Miki T, Morimoto S, Kumahara Y, Mori H, et al. Serum ratio of triiodothyronine to thyroxine and TBG and calcitonin concentrations in Graves’ disease and destruction induced thyrotoxicosis. J Clin Endocrinol Metab. 1981;53(1): 113-16. 10.1210/jcem-53-1-113 6165731

[16] Shigemasa C, Abe K, Taniguchi S, Mitani Y, Ueda Y, Adachi T, et al. Lower serum free thyroxine (T4) levels in painless thyroiditis compared with Graves’ disease despite similar serum total T4 levels. J Clin Endocrinol Metab. 1987;65(2):359-63. 10.1210/jcem-65-2-359 3110204

[17] Izumi Y, Hidaka Y, Tada H, Takano T, Kashiwai T. Simple and practical parameters for differentiation between destruction induced thyrotoxicosis and Graves’ thyrotoxicosis. Clin Endocrinol (Oxf). 2002;57(1):51-8. 10.1046/j.1365-2265.2002.01558.x 12100069

[18] Bogazzi F, Bartalena L, Brogioni S, Burelli A, Manetti L, Tanda ML, et al. Thyroid vascularity and blood flow are not dependent on serum thyroid hormone levels: studies in vivo by color flow Doppler sonography. Eur J Endocrinol. 1999;140(5):452-56. 10.1530/eje.0.1400452 10229913

[19] Kurita S, Sakurai M, Kita Y, Ota T, Ando H, Kaneko S, et al. Measurement of thyroid blood flow area is useful for diagnosing the cause of thyrotoxicosis. Thyroid. 2005;15(11):1249-52. 10.1089/thy.2005.15.1249 16356088

[20] Smith JR, Oates E. Radionuclide imaging of the thyroid gland: Patterns, pearls and pitfalls. Clin Nucl Med. 2004;29(3):181-93. 10.1097/01.rlu.0000114530.12565.5b 15162989

[21] Saleh A, Cohnen M, Furst G, Godehardt E, Modder U, Feldkamp J. Differential diagnosis of hyperthyroidism: Doppler sonographic quantification of thyroid blood flow distinguishes between Graves’ disease and diffuse toxic goitre. Exp Clin Endocrinol Diabetes. 2002;110(1):32-6. 10.1055/s-2002-19992 11835123

[22] Kurita S, Sakurai M, Kita Y, Ota T, Ando H, Kaneko S, et al. Measurement of thyroid blood flow area is useful for diagnosing the cause of thyrotoxicosis. Thyroid. 2005;15(11):1249-52. 10.1089/thy.2005.15.1249 16356088

[23] Ota H, Amino N, Morita S, Kobayashi K, Kubota S, Fukata S, et al. Quantitative measurement of thyroid blood flow for differentiation of painless thyroiditis from Graves’ disease. Clin Endocrinol (Oxf). 2007;67(1):41-5. 10.1111/j.1365-2265.2007.02832.x 17437515

[24] Uchida T, Takeno K, Goto M, Kanno R, Kubo S, Takahashi S, et al. Superior thyroid artery mean peak systolic velocity for the diagnosis of thyrotoxicosis in Japanese patients. Endocr J. 2010;57(5):439-43. 10.1507/endocrj.k09e-263 20513982

[25] X, Chen L, Li L, Wang Y, Wang YO, Zhou L, et al. Peak Systolic Velocity of Superior Thyroid Artery for the Differential Diagnosis of Thyrotoxicosis. PLoS One. 2012;7(11): e50051. 10.1371/journal.pone.0050051 PMC3500337 23166817

[26] Hari Kumar KV, Vamsikrishna P, Verma A, Muthukrishnan J, Rayudu BR, Modi KD. Utility of Colour Doppler Sonography in Patients with Graves’ disease. West Indian Med J. 2009;58:566. 20583684

[27] Ueda M, Inaba M, Kumeda Y, Nagasaki T, Hiura Y, Tahara H, et al. The significance of thyroid blood flow at the inferior thyroid artery as a predictor for early Graves’ disease relapse. Clin Endocrinol (Oxf). 2005;63(6):657-62. 10.1111/j.1365-2265.2005.02397.x 16343100

[28] Baldini M, Castagnone D, Rivolta R, Meroni L, Pappalettera M, Cantalamessa L. Thyroid vascularization by color doppler ultrasonography in Graves’ disease. Changes related to different phases and to the long-term outcome of the disease. Thyroid. 1997;7(6):823-28. 10.1089/thy.1997.7.823 9459623

